# Pushing the frontiers of military medical excellence: updates, progress and future needs

**DOI:** 10.1186/s40779-022-00388-x

**Published:** 2022-06-10

**Authors:** Jun Jie Seah, De-Yun Wang

**Affiliations:** 1 Department of Otolaryngology, Yong Loo Lin School of Medicine, National University of Singapore, Singapore, 119228 Singapore; 2 Infectious Diseases Translational Research Programme, Yong Loo Lin School of Medicine, National University of Singapore, Singapore, 119228 Singapore

**Keywords:** *Military Medical Research*, Military medicine, Clinical medicine, General medicine, Basic science, Coronavirus disease 2019 (COVID-19), Post-traumatic stress disorder (PTSD)

## Abstract

Since its establishment in 2014, *Military Medical Research* has come a long way in becoming a premier journal for scientific articles from various different specialties, with a special emphasis on topics with military relevance. The field of military medicine may be obscure, and may not be readily encountered by the typical clinician on a day-to-day basis. This journal aims not only to pursue excellence in military research, but also to keep current with the latest advancements on general medical topics from each and every specialty. This editorial serves to recap and synthesize the existing progress, updates and future needs of military medical excellence, discussing foremostly the unique traits of literature published in this journal, and subsequently presenting the discourse regarding wartime and peacetime medicine, the role of the military in a public health emergency, as well as wound healing and organ regeneration. Special attention has been devoted to military topics to shed light on the effects of Chemical, Biological, Radiological and Explosive warfare, environmental medicine and military psychiatry, topics which rarely have a chance to be discussed elsewhere. The interconnectedness between military combat and soldier physical and mental well-being is intricate, and has been distorted by pandemics such as coronavirus disease 2019 (COVID-19). This journal has come a long way since its first article was published, steadily contributing to the existing knowledge pool on general medical topics with a military slant. Only with continuous research and sharing, can we build upon the work of the scientific community, with hopes for the betterment of patient care.

## Background

Ever since its inception in 2014 when its inaugural manuscript was published, *Military Medical Research* has continued to publish a plethora of scientific articles contributing to the knowledge base of the scientific community, ranging from case reports to full-fledged practice-changing clinical practice guidelines (CPGs). As a peer-reviewed open access journal, *Military Medical Research* boasts a wide range of research articles, comprising basic science, clinical medicine, and military medicine. Indeed, what sets this journal apart from other general medical journals is its military slant. Compared to other specialties, military medicine can come across as obscure or esoteric to many, and may be unfamiliar to many scientists or clinicians, given that such a subject matter is not encountered on a day-to-day basis. Nonetheless, while this journal maintains its focus on military medicine, *Military Medical Research* also keeps up-to-date with the current medical frontier, accepting original high-quality clinical research articles from various specialties, basic science research and even CPGs. Just recently, a CPG for transurethral plasmakinetic resection of prostate for benign prostatic hyperplasia has been published, building upon its previous 2018 version with newer direct evidence, making evidence-based recommendations for perioperative and postoperative management. Another CPG also newly published summarised evidence-based recommendations for granulomatous lobular mastitis (GLM), which is helpful for a rare disease that previously had no unified guidelines. Recent literature suggested a possible role of *Corynebacterium* infection in the disease process of GLM, providing interesting insights into ever emerging topics.

At the time of writing, *Military Medical Research* has amassed a total of 355 articles (excluding corrections or errata), comprising 116 military-related articles, and 239 non-military articles. Non-military topics still form the majority of articles in *Military Medical Research*, giving it a strong footing in keeping current with medical knowledge.

**Fig. 1:**
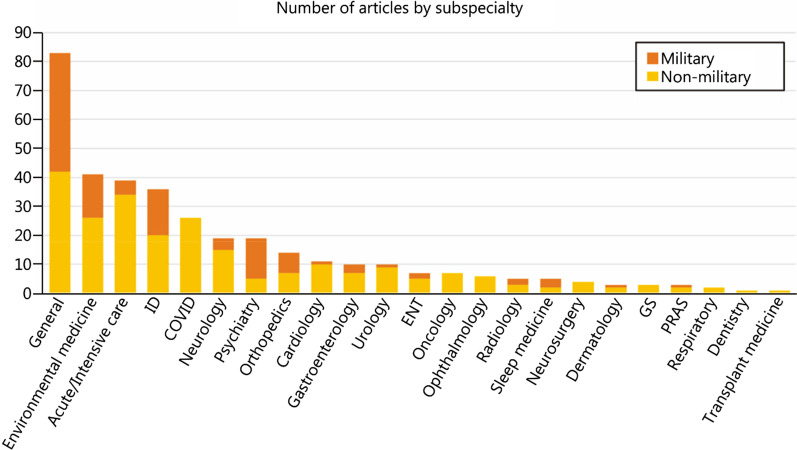
Distribution of articles by various specialties and military relevance. ENT ear, nose and throat, GS general surgery, PRAS plastic, reconstructive and aesthetic surgery

Upon further scrutiny, many various specialties have been included for publication over the past few years, comprising various general and sub-specialized medical and surgical specialties, as well as special topics like coronavirus disease 2019 (COVID-19, Fig. 1). A diverse mix of articles not only keeps readers engaged, but also encourages authors to explore widely.

**Fig. 2:**
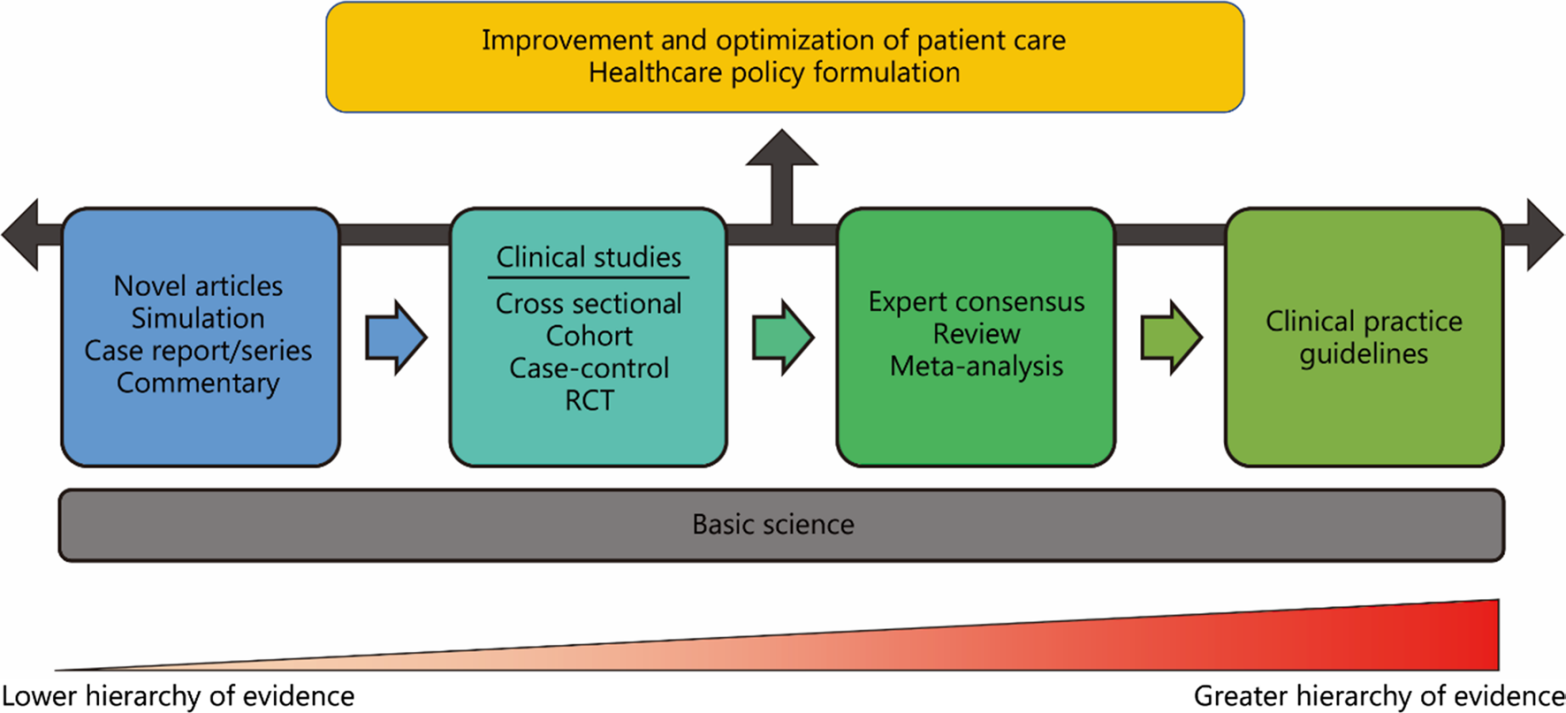
Schematic diagram depicting how each article type builds on the existing knowledge of those before it, with accumulating information leading to greater impact on clinical care. RCT randomised control trial

The different types of articles contribute to the improvement and optimization of patient care in their own unique way. Indeed, while different article design types hold different levels in the hierarchy of evidence, no single article design type takes precedence over another. Every article design focuses on a particular area of knowledge that is necessary for further studies to build upon, and contributes to the existing abundance of clinical knowledge. Eventually, the aim is for betterment of patient care and outcomes (Fig. 2).

**Fig. 3:**
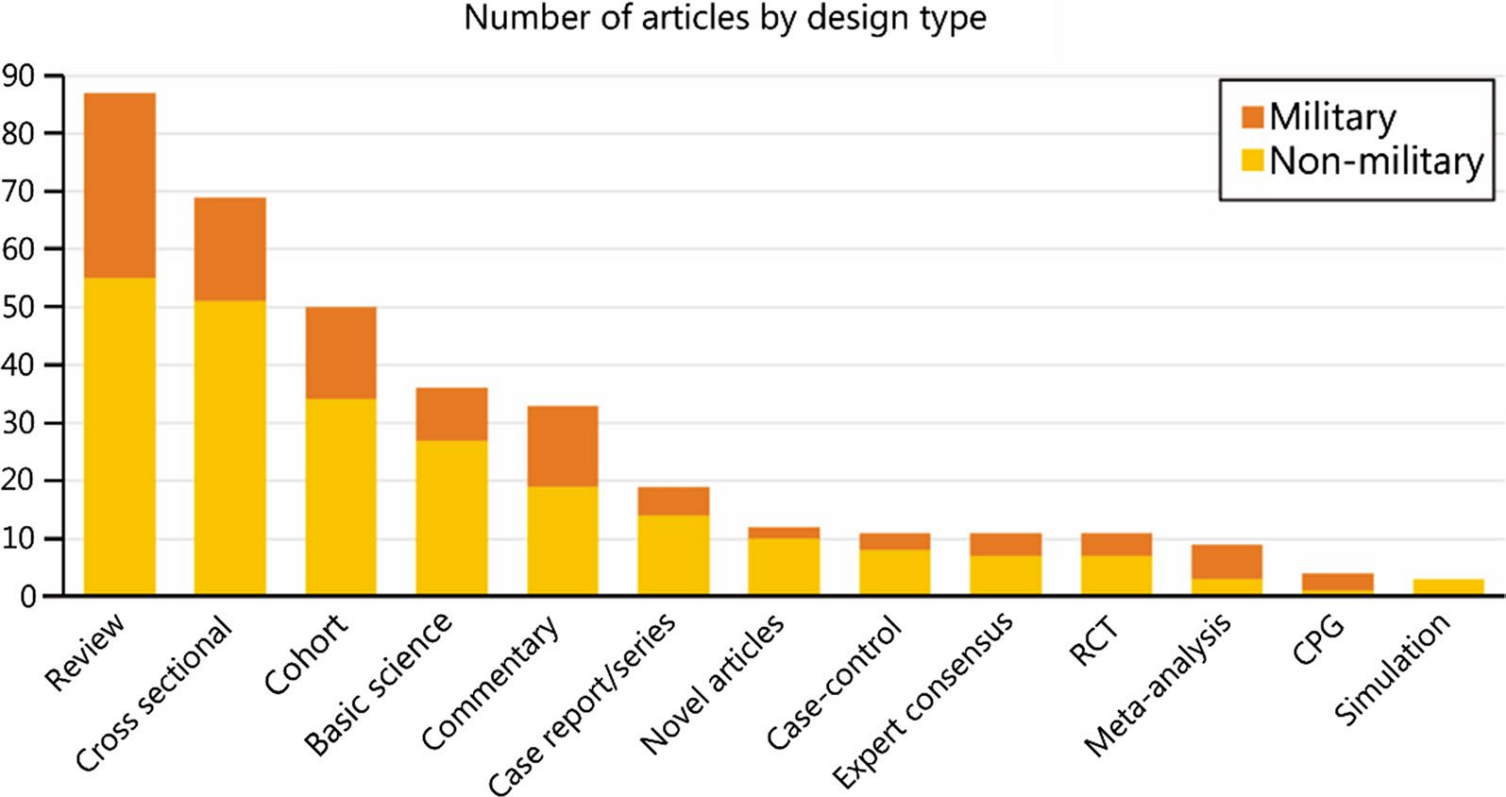
Distribution of article design type and its military relevance. RCT randomised control trial, CPG clinical practice guideline

*Military Medical Research* encompasses all different design types in both military and non-military related articles (Fig. 3). Majority of articles published consisted of review articles, followed by cross-sectional, and cohort studies. Non-military articles generally outnumbered military articles across the various article design types.

Thematic focus is also a feature of *Military Medical Research*. Call for articles attempt to encourage authors to contribute papers pertaining to existing hot topics where medical knowledge is constantly evolving (Table 1). Themes are widely diversified, and comprise medical research from different ends of both military and non-military spectrums. Topics such as altitude medicine, war-related injuries and post-traumatic stress disorder (PTSD) alluding to military psychiatry cannot be ignored in the field of military medicine. On the other hand, topics discussing tissue engineering, microbiome, and COVID-19 are geared towards peacetime medical operations. Themes can span both categories such as the spectrum of sepsis to septic shock, where several high-quality articles with a respectable number of citations have been published, including a basic science article that discussed the role of the IL-33-ST2 axis in sepsis, as well as a meta-analysis that synthesized the existing literature comparing norepinephrine with vasopressin in patients with septic shock. Overall, the varied nature of this journal not only keeps it an interesting read, but it is also pitched to both the generalist and specialist for a one-stop journal for up-to-date knowledge.

**Table 1:** Thematic call for articles over the years in *Military Medical Research*

Year	Theme
2014	Current clinical research progress on high altitude medicine
2016	Sepsis
2017	Health concerns of war-related ankle and foot injuries Post-traumatic stress disorder (PTSD): biopsychosocial translational research and everyday practice
2018	Sepsis and septic shock
2020	Diagnosis and treatment of heat stroke Tissue engineering and regenerative medicine Severe torso trauma: epidemiology, diagnosis, rescue and management
2021	Microbiome Coronavirus disease 2019 (COVID-19)

## Military focus

### Principles of military medicine

Alongside the evolution of modern warfare, healthcare in the military setting has also seen numerous advancements that deserve attention. Resources that have been dedicated to such topics are paramount for us to attain deeper insights into military medicine. This editorial hence presents a timely opportunity to revisit the tenets of military medicine, and understand its importance despite being a relatively esoteric subspecialty.

One way to understand the scope of military medicine would be to consider the dichotomy of peacetime and wartime settings. In the former, a good understanding of civil emergency management is necessary. The principles of mitigation, preparedness, response, and recovery, are tenets that stood the test of time, and can guide healthcare professionals amidst the chaos of a mass casualty scenario. A study has comprehensively described the organization and implementation of mass medical rescue after two earthquakes in Wenchuan of Sichuan Province and Yushu of Qinghai Province. The principles of organizing, coordinating and participating in an efficient and evidence-based medical rescue effort were recounted, resulting in formidable casualty outcomes despite the large-scale devastating nature of the natural disasters. Indeed, sharing of experiences amongst the scientific community results in a win–win situation where learning can take place without repeatedly suffering the wreckage of the catastrophe itself.

In the setting of wartime medicine, a good grasp of the principles of mass casualty triage, disaster management, and damage control resuscitation (DCR) are integral to minimize battle-related morbidity and mortality. The latter has been discussed extensively not only the pathophysiology governing the need for DCR, but also affords statistical data in the context of blast injuries, and incorporating management recommendations in both military and non-military settings. Resource allocation becomes an important doctrine, given that casualties may overwhelm the treatment capacity of the supporting medical force, necessitating accurate and reliable triage to maximize survivors. A study proposed and successfully tested out a novel triage and medical evacuation system for naval warfare, helping to combat the limitations of existing triage systems and improve triage efficiency. In the pre-hospital setting, techniques of Tactical Combat Casualty Care are also heavily emphasized and taught to medics entering the combat arena. The three phases of care under fire, tactical field care, and combat casualty evacuation care are protocolized in detail by Butler Jr et al. [1]. There is an emphasis on advanced trauma life support, to cater to the overwhelming number of traumatic injuries expected in war. Injuries such as gunshot wounds, penetrating thoracoabdominal trauma and burns take centre stage in the range of injuries faced by military medical personnel. These aforementioned topics have been heavily discussed in *Military Medical Research*.

### Special fields of military medicine

#### Chemical, Biological, Radiological and Explosive (CBRE) warfare

CBRE warfare is rarely encountered during peacetime, as it is difficult to realistically simulate for training purposed. A study explored the challenges with emergency medical preparedness and response in CBRE attacks, and proposed appropriate countermeasures based on prior real-life experiences. An interesting strategy utilizes a professional multidisciplinary consulting team comprising physicians, nuclear physics and chemical experts to efficiently tackle such unique threats. It has also been discussed that CBRE warfare with a special focus on radiological dispersal devices (also known as “dirty bomb”). The danger of a dirty bomb detonation is attributed not only to its explosive effect causing blast injury, but also to its area denial effect, where the radioactive contamination mandates quarantine and intensive large-scale clean-up, resulting in marked economic as well as short- and long-term health effects.

#### Environmental medicine

The effects of the environment on the health of soldiers most definitely cannot be ignored. Especially in tropical climates, high temperatures can impact the physical and mental well-being of soldiers, regardless of peacetime or wartime activities. Heat-related illnesses, such as mild heat illness, heat injury, or heat stroke, refer to the spectrum of physiological disturbances that occur when the body is exposed to high environmental temperatures, with consequent deleterious sequelae on the cardiovascular, gastrointestinal, hepatobiliary, musculoskeletal, and central nervous systems. Under thermal stimulation, pathological decompensation of bodily systems can occur, consequently leading to systemic inflammation and multi-organ failure. Heat acclimatization may hence attenuate these effects by initiating physiological and behavioral adaptations to protect thermal homeostasis, improve performance and reduce heat illness risk. A study developed a 47-point scoring system to assess the severity of exertional heat stroke (EHS), with variables comprising clinical parameters, laboratory tests, and acute gastrointestinal injury classification. Their score can predict mortality from EHS, performing better than the Acute Physiology and Chronic Health Evaluation II and Sequential Organ Failure Assessment scores in this aspect. On the other end of the spectrum, cold injury is not as frequently encountered and researched upon, and manifests mainly in temperate climates. As discussed at length in an expert consensus statement regarding the epidemiology, prevention, diagnosis and treatment of cold injuries, which have a much higher incidence in military than the civilian setting. Many lessons can be learnt from the experiences of environmental injury, and it is important to remain cognizant of its effects in the military and civilian settings.

#### Military psychiatry

Military psychiatry is also an emerging field in the wartime setting. With reference to Fig. 1, psychiatry is one of the few specialties where military articles outnumbered non-military ones in *Military Medical Research*, depicting the increasing emphasis and importance of military psychiatry that has become an invaluable and intangible effect of war. The phenomenon of combat stress reaction (CSR) refers to labile polymorphic manifestations affecting cognition, affect, and behavior during battle [2]. Risk factors for the development of CSR non-exhaustively include older soldiers, lack of physical fitness, lower educational level, married individuals, lower rank and experience, and those with a recent birth or death in the family. In contrast, PTSD is characterized by a constellation of re-experience and avoidance symptoms after exposure to a traumatic event, and can potentially be chronically debilitating. A study on 1730 war veterans from Pennsylvania found that significant predictors of PTSD included female sex, high combat exposure, history of concussion, high stressful events within the past year, high lifetime trauma exposure, low social support, and low social capital. Interestingly, serving on multiple tours was a protective factor for PTSD. It is important to distinguish between CSR and PTSD, as the latter can be easily undetected and lead to long-lasting psychological sequelae. In addition, the effects of military experiences on dementia risk have been reviewed, which found that military deployment, traumatic brain injury (TBI), PTSD, and poor sleep all contribute to dementia development, with the strongest link arising from TBI. The effect of the military on the mental health of soldiers is not a temporary one, but can have long-term deleterious effects even long after they have left the military, impacting on the quality of life. To tackle the negative effects of sleep deprivation or poor sleep quality on the mental health well-being of soldiers, the chronotype profile of U.S. sailors was studied, and aligning military schedules with endogenous circadian rhythms was suggested to optimize performance. On the other hand, it found that quetiapine monotherapy can increase engagement in trauma-focused psychotherapy, given that fluoxetine, the alternative pharmacologic treatment for PTSD, has an extended onset of action and can exacerbate post-concussion syndrome in mild TBI. Despite military psychiatry being an established field, there leaves much more knowledge to be discovered in this ever-evolving specialty, which is only made possible with continued research.

## Medical support in a public health emergency: a word on COVID-19

The response to infectious disease crisis represents one of the major tasks of the military during peacetime medical operations. The COVID-19 pandemic has, without a doubt, revolutionized the focus of the scientific community since end-2019. Over the past two years, COVID-19 has not only been the talk of the town, but many scientific articles, be it basic scientific papers, clinical studies or trials, or even case reports of unique phenomena, have sprung up and populated the pages of many medical journals. The dynamic nature of articles published about COVID-19 since the start of the pandemic reflects how our understanding of the disease evolves as we learn more about it.

### Basic science and advancements

Basic science articles form the foundation and basis for other clinical studies to build upon, and are integral in the comprehensive understanding of the pathobiology of disease, especially in the context of COVID-19 virology. A study delved right into the essence of basic science in a laboratory study on human lung epithelial cell lines, explaining how retinoic acid-inducible gene I binds the 3’ untranslated region of the severe acute respiratory syndrome coronavirus 2 (SARS-CoV-2) RNA genome via its helicase domains, preventing viral RNA replication independently of interferons, and can also restrain full-length angiotensin-converting enzyme 2 (ACE2) expression and consequently cellular entry by SARS-CoV-2. Neighboring cell–cell fusion which produces multinucleated syncytia is implicated in the pathogenesis of COVID-19, and that such syncytia were readily detected in the post-mortem lung tissues of patients with COVID-19 alongside extensive damage in tissue structures. These syncytia also facilitated virus spread via the cell-to-cell route, shielded from the extracellular antibodies that hinder cell-free transmission.

Another systematic review on the topic of neutralizing antibodies in COVID-19 infection, discusses not only the pathomechanism of how neutralizing antibodies interfere with the virulence of the SARS-CoV-2 virus, but also explores the differential antibody response in mild and severe COVID-19 infection, expounding on how the rapidity of neutralizing antibody production has implications on mortality. Indeed, a rooted understanding of virology serves as the foundation for further research on COVID-19, including vaccine development and pharmacological treatment.

An interesting review providing a timely update on the COVID-19 status was published in March 2020, just shortly after the World Health Organisation declared COVID-19 a pandemic. This heavily-cited article shared comprehensive insights on various aspects of the disease, ranging from basic virology and mechanism of infection, to practical tips on clinical diagnostic and management recommendations. Even with such a recent disease that broke out not long ago, the variety in research articles published in *Military Medical Research* is limitless, exemplifying its role as a general medical journal hub for knowledge exchange.

### Effect on organ systems

It is not unexpected that COVID-19, being a new disease, has many unknown sequelae on various different organ systems apart from its primary pathology of causing respiratory tract infection. *Military Medical Research* has received various articles describing the manifestations of COVID-19 on different systems. From the hematological point of view, COVID-19 can cause coagulation dysfunction as a result of systemic inflammatory reactions leading to microvascular damage, abnormal coagulation system activation, manifesting pathologically as a small-vessel vasculitis and extensive microthrombosis. COVID-19 also causes hepatic injury, manifesting primarily as deranged liver function tests. Mechanisms to account for this include direct hepatocyte injury, drug-induced liver injury, hypoxic-ischemic microcirculation disorder, or underlying liver diseases. In addition, patients with severe COVID-19 had significantly higher liver enzyme elevations compared to mild cases, seeming to suggest a positive relationship between COVID-19 severity and the extent of liver injury. COVID-19 also affects the gastrointestinal system, manifesting non-specifically as anorexia, nausea, vomiting, and diarrhea, supported by positive results of gastrointestinal tract and stool RNA samples. In several studies, the occurrence of diarrhea ranges from 2.0 to 49.5% [3]. This may be associated with the fact that ACE2, the binding receptor for SARS-CoV-2, is highly expressed in the ileum and colon. In a cross-sectional study in China involving 187 patients with confirmed severe COVID-19 infection, it is purported that the SARS-CoV-2 virus neuroinvades via olfactory sensory neurons, colonizing olfactory bulb tissue. Increased expression of ACE2 in the nasopharynx may lead to an increased risk of olfactory and taste symptoms. The authors also propose using olfactory or taste disturbance as an early-warning symptom to screen for patients potentially infected with COVID-19, especially in the absence of rhinitis. The consequences of COVID-19 infection on various organ systems are aplenty, and it is prudent to be aware of such unique manifestations when encountering patients with COVID-19 infection with associated atypical symptoms.

### Prognostication of COVID-19

Prognostication of COVID-19 patients is another interesting field of study that has garnered research attention. Presenting complaints range from completely asymptomatic with a positive diagnostic SARS-CoV-2 polymerase chain reaction swab test, to full-blown pneumonia with acute respiratory failure that can be life-threatening. Prognostication of infected patients plays an important role in determining which patients will require closer monitoring and intervention, as well as selecting patients who will benefit from further drug therapy. A Chinese study on 2541 confirmed COVID-19 patients found that older age, lymphopenia, respiratory rate ≥ 30/min, and high IL-6 levels were independent high-risk factors associated with poor prognosis. A study also developed a nomogram which serves as a quick visual aid to estimate the risk of fatal outcomes. It has been proposed by a workflow for risk stratification of COVID-19 patients via simple scoring of clinical, blood and imaging tests. Its efficacy, however, has yet to be tested in a patient population, and may be limited by the need for routine chest CT. C5178a and A249d mitochondrial DNA (mtDNA) variants are found to be associated with a reduced risk of severe COVID-19, while the A4833G, A4715G, T3394C, and G5417A variants were related to an increased risk of severe COVID-19. Testing for common mtDNA variants via a simple blood test may eventually play a role in stratifying patients infected with COVID-19, providing an additional triage tool to enhance clinical assessment.

### Treatment and preventive measures

The current research on COVID-19 treatment is heterogenous, with many proposed treatment strategies with claims of effectiveness. A review has comprehensively synthesized the current evidence pertaining to investigational therapies for COVID-19, and their role as adjuncts to standard supportive care. Given that there is so much more yet to be discovered about COVID-19, articles like such efficiently centralize the myriad of existing data, providing new insights by harnessing the findings of existing knowledge.

As the COVID-19 pandemic progresses, finding a medical cure may be important, but a long-term sustainable solution to combat such spread involves preventive measures. A study proposed a novel bionic nanoparticle vaccine, which has the benefits of desirable biocompatibility, as well as being able to simulate the whole virus structure, including its infection process, to efficiently trigger antibody production. With such advances, the ability to eventually develop “universal” influenza vaccines is promising. For patients who fall through the cracks, early detection can serve as the next line of defense. Rapid diagnostic kits such as the antigen rapid test or the SARS-CoV-2 real-time reverse-transcription polymerase chain reaction test may yield false positive results. Adedokun described the clinical entity termed “silent hypoxemia” where patients can be completely asymptomatic or minimally symptomatic, but subsequently deteriorate insidiously without warning, sometimes to the extent of progression to respiratory failure without the warning of respiratory distress. Pulse oximetry can screen asymptomatic patients with low oxygen saturations for further workup, which additionally has the benefit of being portable and easily administered.

### Relationship between COVID-19 and the military

Military hospitals have demonstrated efficiency in combating the current COVID-19 pandemic via rapid protocol planning and standard operating procedures. Many approaches can be learned from their experiences responding to such a public health crisis, which play an integral role by contributing to the currently existing knowledge pool, providing new insights into the field of pandemic readiness and response. Scientific research on military medicine has also highlighted the importance of medical support in peacetime military operations. How we can further extend the scope of medical support afforded by the military in a pandemic setting is an important topic that requires further research. *Military Medical Research* examines these issues via its repertoire of publications, ensuring a diversity of trending scientific knowledge resources.

A study described their experience with an outbreak of *Mycoplasma pneumoniae* in a Chinese military academy. They reasoned that the high-intensity training and psychological stress decrease immunity, and environmental factors such as climate, high-density residences, and non-ventilated rooms accounted for the outbreak. Indeed, the congregation of military service personnel in a densely packed setting coupled with regular and frequent close interpersonal interaction acts as a catalyst for disease spread.

Interestingly, a study described the evolving role of the military in pandemics, such as COVID-19, and how the austere environment creates challenges when the military is deployed to assist in pandemic medical efforts.While their medical facilities system is meant for triage and stabilization of battlefield injuries, it was not designed to provide potentially long-term respiratory support to large acute volumes of patients. They drew parallels to the Spanish Influenza epidemic of 1918 (amidst World War I) and the typhoid fever epidemic of 1898 (amidst the Spanish-American War), where the infective pathogen led to greater fatalities of military personnel compared to combat casualties. Infectious diseases can be sufficiently devastating to affect the trajectory of war, and remind us never to underestimate the lethality of contagious pathogens.

War and infectious diseases are intimately intertwined. War affects disease in that it provides a conducive environment with highly clustered soldiers who interact closely on a day-to-day basis, providing ease of human-to-human transmission. The physical and psychological degradation of war on the human body may also possibly undermine the immune system, making one more susceptible to contracting disease. Disease also affects war, by increasing the already dismal casualty numbers sometimes more than what is caused by combat alone, or via strategies used to incorporate biological agents as weapons to gain an upper hand against the enemy. Indeed, the lethal, invisible and covert nature of infectious diseases makes it a difficult enemy to identify, until its disastrous effect on the human body manifests, which by then may be too late.

## Wound healing and organ regeneration

### Advances in wound healing

Wound healing is a unique genre relevant to both military and non-military medicine. Surviving casualties of non-lethal injuries eventually require definitive wound care, a potentially arduous long-drawn process fraught with threats of complications. In the non-military setting, wound healing is also a widely-discussed topic, inevitably encountered in patients undergoing surgery, or in patients suffering from vasculopathy with a slow-healing foot ulcer, for instance. Albeit to a smaller extent, the civil setting is also not exempt from high-energy blast energy injuries. A database analysis on 2098 explosions over 18 years found a decreasing but significant trend in the number of explosions in China. Casualties importantly lack training and personal protective equipment, and are thus prone to more severe injuries compared to soldiers. Given the ubiquitous nature of penetrating or blast injuries in both the military and non-military settings, a firm grasp of the principles of wound healing is necessary. For optimal wound healing to take place, treatment should focus on optimization at the cellular level, such as by removal of reactive oxygen species, before enhancing the wound bed via chemical or surgical debridement.

A few authors investigated the effect of novel methods for wound healing in animal studies. A study that was performed in Bama pigs and discovered that recombinant human epidermal growth factor at a concentration of 10 ng/ml can promote the proliferation and migration of epithelial cells and fibroblasts to the greatest extent, and can be further enhanced by vacuum sealing drainage. The use of LL-37 peptide has been confirmed to promote wound healing by incorporating it into chitosan hydrogel, improving its stability in the wound environment, thus improving the healing of pressure ulcers in a mouse model. The technique of ex vivo limb perfusion via a hypothetical experiment and prototype has been discussed, which can be applied hypothetically in the evacuation and treatment of a fictive patient. Novel studies are important in providing a stepping stone for further research, especially in a field still under active exploration.

Bioelectrical impedance analysis-guided fluid resuscitation in post-traumatic open abdomen patients managed to achieve a higher primary fascial closure rate and fewer complications compared to the traditional resuscitation strategy. In the military setting, the role of platelet-rich plasma (PRP) on military drill injury has been reviewed, as well as discussed the pros and cons and surrounding controversies of PRP therapy. While there seems to be existing limitations to the use of PRP, the minimally-invasive nature of the treatment and its potential benefits make it an attractive option in the therapeutic armamentarium. An orthopedic surgeon also analyzed his experience with patients requiring soft tissue coverage using pedicled flap in the combat arena. The nuances of wound reconstruction in the military setting are explored, where emergency soft tissue cover is often required, but evacuation to a tertiary hospital with plastic surgery expertise may not be possible for various reasons.

### Advances in organ regeneration

It is also pertinent to discuss the progress on organ regeneration, given that traumatic solid organ injury is a common finding in the wartime setting. Toll-like receptor 5 signalling can positively regulate and promote liver regeneration by enhancing proinflammatory responses in a mouse model, potentially opening up therapeutic applications for partial hepatectomy patients. A study performed experiments in a Wistar albino rat model, and showed that the use of autologous omentum in addition to primary repair for traumatic kidney injury attenuated the extent of inflammation and granulation, which may facilitate healing of kidney injury and reduce fibrosis and future functional loss. Progenitor cells within the omentum can migrate to damaged tissues and aid in the regeneration process. Neuroplasticity can also manifest in the central nervous system post-injury via the mechanism of cortical remapping, where functions lost as a result to central nervous system damage is remapped to another part of the cerebral cortex. In the repair of spinal cord injury, hydrogels have mechanical properties that mimic the extracellular matrix of the spinal cord, providing a scaffold for axonal growth and neuronal formation, removal of inflammatory cells and factors, eliminating spinal cord cyst formation, and inhibition of glial scarring. Advances in organ regeneration and healing can revolutionize treatment modalities and optimize patient care. *Military Medical Research* provides a unique perspective on the ongoing discourse on wound healing and organ regeneration by providing an additional emphasis from a military standpoint, complementing experiments and studies performed in the civil setting.

## Future needs

While *Military Medical Research* has met many of the objectives it set out to achieve ever since its establishment in 2014, this editorial serves as a timely opportunity to consolidate the journal’s achievements thus far, and propose directions for future scientific work. New knowledge pertaining to both peacetime and wartime military medicine are still paramount in the journal’s aim to promote the global development and progress of military medicine. Fresh innovative ideas regarding the improvement of military medical efficiency, technological healthcare advancements, as well as soldier performance maximization will definitely contribute to the forefront of military medical scientific knowledge. At the same time, general medical research articles are equally important, as they serve to keep everyone current about the latest cutting-edge technology and clinical data. The interaction between military and civilian medicine may not be apparent at first, but upon close scrutiny, many lessons that transpired from both military wartime and peacetime operations can be applied in the civilian context to optimize the delivery of healthcare.

## Conclusions

*Military Medical Research* has come a long way since its establishment in 2014. With its acceptance of a wide range of articles, it stands the journal in good stead as a general medical journal with a military slant, standing out uniquely by discussing topics not be frequently seen in other conventional medical research journals, which can bring interesting insights and perspectives to various topics. Only with continued support by clinicians, researchers, and scientists from all fields of medicine, can medical knowledge be freely shared and built upon one another, allowing the frontiers and boundaries of medicine to be stretched.

## Data Availability

Not applicable.

## Abbreviations

CBRE: Chemical, Biological, Radiological and Explosive
COVID-19: Coronavirus disease 2019
CPG: Clinical practice guideline
CSR: Combat stress reaction
DCR: Damage control resuscitation
EHS: Exertional heat stroke
GLM: Granulomatous lobular mastitis
mtDNA: Mitochondrial DNA
PRP: Platelet-rich plasma
PTSD: Post-traumatic stress disorder
SARS-CoV-2: Severe acute respiratory syndrome coronavirus 2
TBI: Traumatic brain injury
