# Correction to: Pushing the frontiers of military medical excellence: updates, progress and future needs

**DOI:** 10.1186/s40779-022-00436-6

**Published:** 2022-12-23

**Authors:** Jun Jie Seah, De-Yun Wang

**Affiliations:** 1 Department of Otolaryngology, Yong Loo Lin School of Medicine, National University of Singapore, Singapore, Singapore; 2 Infectious Diseases Translational Research Programme, Yong Loo Lin School of Medicine, National University of Singapore, Singapore, Singapore

**Correction to: Military Medical Research (2022) 9:27** 10.1186/s40779-022-00388-x

Following publication of the original article [1], it was found that the article version is incorrect, the correct version article has been updated in the original paper.
